# Serum Malondialdehyde-Oxidized Low-Density Lipoprotein Level Is Associated with Arterial Stiffness by Cardio-Ankle Vascular Index in Coronary Artery Bypass Graft Patients

**DOI:** 10.3390/jcm12134191

**Published:** 2023-06-21

**Authors:** Ting Hung, Jin-You Jhan, Jian-Hong Lin, Kun-Ta Yang, Bang-Gee Hsu, Jui-Chih Chang

**Affiliations:** 1 Division of Cardiovascular Surgery, Department of Surgery, Hualien Tzu Chi Hospital, Buddhist Tzu Chi Medical Foundation, Hualien 97002, Taiwan; dinghung16@gmail.com (T.H.); lawj4068@gmail.com (J.-Y.J.); 2 Division of Experimental Surgery, Department of Surgery, Hualien Tzu Chi Hospital, Buddhist Tzu Chi Medical Foundation, Hualien 97002, Taiwan; 101327102@gms.tcu.edu.tw; 3 School of Medicine, Tzu Chi University, Hualien 97004, Taiwan; ktyang@mail.tcu.edu.tw; 4 Division of Nephrology, Department of Internal Medicine, Hualien Tzu Chi Hospital, Buddhist Tzu Chi Medical Foundation, Hualien 97002, Taiwan

**Keywords:** malondialdehyde-oxidized low-density lipoprotein, cardio-ankle vascular index, arterial stiffness

## Abstract

A high malondialdehyde-oxidized low-density lipoprotein (MDA-oxLDL) level is associated with atherosclerotic cardiovascular diseases and major adverse cardiovascular events. A higher cardio-ankle vascular index (CAVI) is independently associated with an increased risk of cardiovascular events, cardiovascular mortality, myocardial infarction, and stroke in patients with cardiovascular risk. Thus, this study aimed to evaluate the relationship between serum MDA-oxLDL levels and CAVI in patients with triple-vessel coronary artery disease who underwent coronary artery bypass graft (CABG) surgery. Fasting blood samples and baseline characteristics were obtained from 88 patients who had undergone CABG. A commercialized enzyme-linked immunosorbent assay was used to measure MDA-oxLDL levels. An automatic pulse wave analyzer was used to measure CAVI values, and each side of CAVI values of ≥9 was designated as arterial stiffness. In total, 47 participants were assigned to the arterial stiffness group. More patients had diabetes mellitus, were older, and had higher serum MDA-oxLDL levels in the arterial stiffness group than in the control group. A multivariate logistic regression analysis disclosed that MDA-oxLDL and diabetes mellitus were independent predictors of arterial stiffness. Moreover, according to the Spearman’s correlation analysis, the serum MDA-oxLDL level was positively associated with both left and right CAVI. Serum MDA-oxLDL levels were positively associated with arterial stiffness in patients who had undergone CABG.

## 1. Introduction

Arterial stiffness is an aging process of the human body that results from combined mechanisms, including endothelial dysfunction, oxidative stress, elastin degradation, collagen deposition, calcium, advanced glycation end-product, and altered vascular smooth muscle tone [1]. Arterial stiffness could result in decreased vascular buffering capacity, increased pulse pressure, and target-organ damage [2]. Arterial stiffness is also related to coronary artery diseases, strokes, chronic kidney diseases, and all-cause mortality [1,3]. Several methods have been used to measure the extent of arterial stiffness, and those commonly used were pulse wave velocity (PWV) and cardio-ankle vascular index (CAVI) [1,4]. The CAVI is a non-invasive diagnostic test that assesses the arterial stiffness from the heart to the ankle [4,5,6]. CAVI is based on the stiffness parameter β, which is, theoretically, independent of blood pressure at the time of measurement [5,6]. Many studies have shown that CAVI is a sign of arteriosclerosis and is related to cardiovascular (CV) disease. In addition, CAVI ≥ 9.0 is proposed as the best cut-off value for predicting CV events [5,6,7].

The oxidized low-density lipoprotein (ox-LDL), a product of the oxidative process in humans, is related to atherosclerotic plaque formation and thrombotic event [8,9]. The serum malondialdehyde-oxidized low-density lipoprotein (MDA-oxLDL), which is a type of ox-LDL with MDA adduction onto its apolipoprotein B, is also related to atherosclerosis, increased risk of an acute coronary syndrome, in-stent restenosis after percutaneous coronary intervention, major adverse CV events, and the need for revascularization [10,11,12,13,14,15]. MDA-oxLDL was also found to be an independent predictive factor of high-risk atherosclerotic plaque [16]. Previous studies have revealed that a high serum ox-LDL level was associated with higher carotid–femoral PWV in older persons, patients with lower extremity atherosclerosis, and healthy individuals, and these results indicate that the ox-LDL level might be associated with arterial stiffness [17,18].

Patients with coronary artery diseases who need surgical revascularization are those who have relatively higher plaque burden, oxidative stress, and CV risks. Comorbidities, such as diabetes mellitus (DM), hypertension, chronic kidney diseases, and arterial stiffness, affect the long-term prognosis of patients who had undergone coronary artery bypass graft (CABG) surgery [19]. Thus, this study aimed to evaluate the relationship between serum MDA-oxLDL levels and CAVI in patients with triple-vessel coronary artery disease who underwent CABG surgery.

## 2. Materials and Methods

### 2.1. Patients

This was a cross-sectional observational study, and participants were recruited from the cardiovascular surgery outpatient department in a single center of Hualien Tzu Chi Hospital, Hualien, Taiwan, between September 2018 and May 2019. There were 88 patients with a history of triple-vessel coronary artery disease who had undergone CABG enrolled in this study. All participants provided written informed consent. Blood samples were taken for laboratory analysis. CAVI and ankle–brachial index (ABI) were measured. Participants were excluded from the study if they had certain medical illnesses, such as malignancies (*n* = 3), acute infection (*n* = 1), heart failure (*n* = 1), amputation (*n* = 3), acute myocardial infarction (*n* = 1), or an ABI < 0.9 (*n* = 15), at the time of enrollment. Patients were regarded as having DM if the fasting plasma glucose was ≥126 mg/dL or were using oral hypoglycemic medications or insulin. Blood pressure (BP) was measured in the morning using standard electronic sphygmomanometers with appropriate cuff sizes after resting for at least 10 min. Systolic BP (SBP) and diastolic BP (DBP) were taken three times at 5 min intervals, and the average values were recorded for the analysis. Hypertension was defined as SBP ≥ 140 mmHg and/or DBP ≥ 90 mmHg in patients who had received antihypertensive medication in the previous two weeks.

### 2.2. Anthropometric Analysis

Anthropometric variables were measured in the morning after overnight fasting. Body weight and height were measured to the nearest 0.5 kg and 0.5 cm, respectively. Body mass index was calculated as weight (kg) divided by height squared (m^2^).

### 2.3. Biochemical Determinations

Fasting blood samples (approximately 5 mL) were immediately centrifuged at 3000× *g* for 10 min. Serum levels of blood urea nitrogen, creatinine, fasting glucose, total cholesterol, triglycerides (TG), high-density lipoprotein cholesterol (HDL-C), and low-density lipoprotein cholesterol (LDL-C) were measured using an autoanalyzer (Siemens Advia 1800; Siemens Healthcare GmbH, Henkestr, Germany). Serum cystatin C levels were measured using a nephelometric Siemens immunoassay (Dimension Vista 1500, Siemens Healthcare GmbH, Henkestr, Germany). The estimated glomerular filtration rates (eGFRs) were determined using a Chronic Kidney Disease Epidemiology Collaboration equation (CKD-EPI Creatinine 2021). Serum MDA-oxLDL levels were measured using a commercially available enzyme-linked immunosorbent assay (Biomedica, Divischgasse, Vienna, Austria). The intra- and inter-assay coefficients of variation were 6.8% and 5.5%, respectively.

### 2.4. CAVI and ABI Measurements

Measurements were performed in a quiet, temperature-controlled room. After 10 min at rest in the supine position, CAVI was measured using the VaSera VS-1000 (Fukuda Denshi Co. Ltd., Tokyo, Japan). Briefly, cuffs were applied to both arms and ankles, with the patient supine and the head held in the midline position. Then, the phonocardiography microphone and electrocardiography electrodes were set up at the designated location. The VaSera VS-1000 measured the brachial and ankle blood pressures, and the ABI and CAVI were automatically calculated. On the basis of an expert consensus from the Vascular Failure Committee in the Japan Society for Vascular Failure, CAVI of ≥9 was a statistically adequate value to indicate individuals with significant arterial stiffness and a high risk of cardiovascular disease [20]. In this study, the arterial stiffness group was defined as those with CAVI values of ≥9, whereas the control group had CAVI values of <9.

### 2.5. Statistical Analysis

The Kolmogorov–Smirnov test was used to examine the normality of continuous variables. According to the results, these continuous data were expressed as the mean ± standard deviation or median with interquartile ranges. To compare the difference in continuous variables between the control and arterial stiffness groups, Student’s independent *t*-test or the Mann–Whitney U test was applied according to the Kolmogorov–Smirnov test, as appropriate. Categorical variables were presented as numbers along with percentages and compared using the chi-squared test. By using multivariable logistic regression analysis, we further assessed variables significantly associated with arterial stiffness, which was defined as CAVI ≥ 9. Factors that showed association with arterial stiffness were used in multivariable logistic regression analysis. A receiver operating characteristic (ROC) curve analysis was used to determine the optimal serum MDA-oxLDL levels indicative of arterial stiffness. Spearman’s rank correlation coefficient was used to analyze the correlation between the clinical variables and CAVI (left and right) and the MDA-oxLDL. IBM SPSS for Windows version 19.0 (IBM Corp., Armonk, NY, USA) was used for all statistical analyses. A *p* < 0.05 was indicative of statistical significance.

## 3. Results

In this study, 47 (53.4%) and 41 (46.6%) patients comprised the arterial stiffness and control groups, respectively (Table 1). Compared with the control group, the arterial stiffness group was older (*p* = 0.004), had a higher percentage of DM (*p* = 0.029), and higher serum MDA-oxLDL levels (*p* < 0.001). Arterial stiffness did not differ significantly by sex or hypertension in this patient group. No significant difference in lipid profiles, fasting glucose, or renal function was found between the groups.

**Figure 1:**
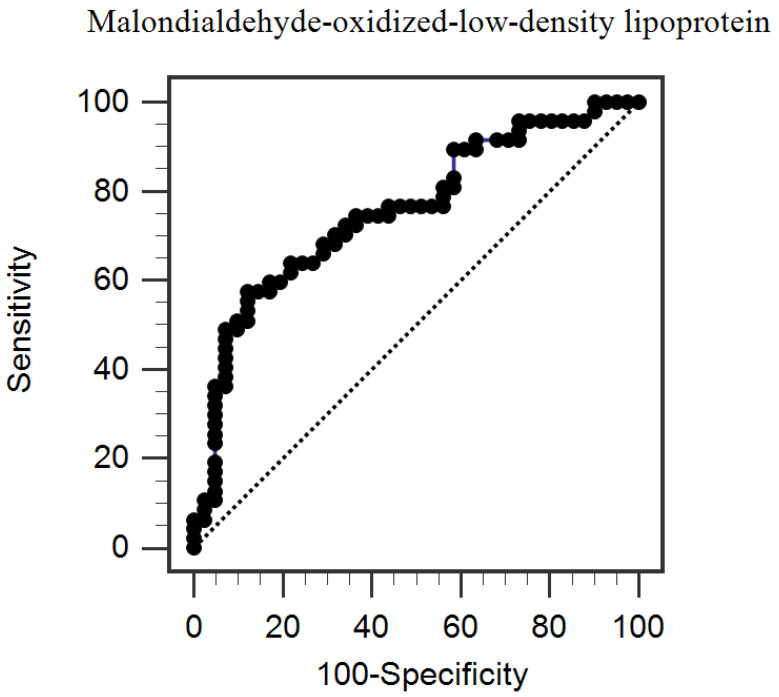
The area under the receiver operating characteristic curve indicates the diagnostic power of serum malondialdehyde-oxidized low-density lipoprotein levels for predicting peripheral arterial stiffness among the 88 coronary artery bypass graft patients. Area under the ROC curve (AUC): 0.755. 95% confidence interval: 0.652 to 0.840. *p* < 0.0001.

After adjusting for factors that showed association with arterial stiffness (*p* < 0.2, female, DM, age, triglyceride, height, BMI, Cystatin C, and MDA-oxLDL from Table 1), the multivariable logistic regression analysis revealed that serum MDA-oxLDL level (odds ratio (OR) 1.511, 95% confidence interval (CI) 1.059–2.158, *p* = 0.023) and DM (OR 3.660, 95% CI 1.121–11.943, *p* = 0.032) were independent predictors of arterial stiffness in patients with triple-vessel coronary artery disease who had undergone CABG (Table 2). To evaluate the accuracy of the serum MDA-oxLDL level as a predictor of arterial stiffness and to find the most appropriate cutoff value, an ROC curve analysis was performed, which showed that the area under the curve was 0.755 (95% CI 0.652–0.840, *p* < 0.0001, Figure 1). The best cutoff value of serum MDA-oxLDL was 2.14 µg/mL, with sensitivity of 57.4% and specificity of 87.8%.

Table 3 shows the correlation between the clinical variables and CAVI (left and right) and the serum MDA-oxLDL level by Spearman’s correlation analysis. Both left and right CAVI showed a significantly positive correlation with serum MDA-oxLDL level (*r* = 0.313, *p* = 0.003 and *r* = 0.348, *p* < 0.001, respectively).

## 4. Discussion

The results of this study revealed that older age, DM, and higher levels of serum MDA-oxLDL were associated with arterial stiffness in patients with triple-vessel coronary artery disease who had undergone CABG. After adjusting for confounding factors, the multivariable logistic regression analysis revealed that MDA-oxLDL levels and DM were independent predictors of arterial stiffness in these patients.

Arterial stiffness increases with age, habits (smoking), and comorbidities, such as hypertension, DM, dyslipidemia, obesity, and sleep apnea [5,7]. Severe arterial stiffness results in systolic hypertension, increased pulse pressure, and decreased coronary perfusion, which further causes myocardial ischemia, ventricular remodeling, and dysfunction [1,2]. Moreover, an increase in pulse pressure increases the transduction of pulsatile flow into the capillaries of target organs, particularly the kidneys and brain, causing end-organ damage [1,2,5].

Since CAVI’s proposal in 2004, many studies have examined its utility as a relatively new method for evaluating arteriosclerotic disease and vascular stiffness [1,4]. CAVI is strongly correlated with the stiffness parameter β, and it is less dependent on blood pressure than PWV [1,4]. The predictive value of CAVI for CV risks has also been established in many studies. In one single-center observational study enrolling 238 patients who had undergone elective CABG, the patients with pathological CAVI (≥9.0) were more likely to develop CV complications and CV death within the 5-year follow-up period [21]. Several studies have pointed out that CAVI is high in patients with CV risk factors, such as hypertension, DM, dyslipidemia, sleep apnea syndrome, and smoking habits [7]. High CAVI was also observed in patients with arteriosclerotic disease, such as coronary artery disease, cerebral infarction, and chronic kidney disease [7]. In one multicenter prospective cohort study enrolling 2932 patients with CV risk, CAVI was associated with the risk of CV death, stroke, all-cause mortality, and heart failure requiring hospitalization [6]. Several treatments (blood pressure control, glucose control, and lipid control) and lifestyle modifications (body weight reduction, smoking cessation, and exercise) reduced CAVI [7]. Thus, identifying those patients with higher risks of developing cardiovascular complications and cardiovascular death is imperative for implementing risk-reduction interventions in clinical practice. Our cohort is composed of patients with triple-vessel coronary artery disease who had multiple risk factors of atherosclerosis and arterial stiffness. The median CAVI values were 8.8 and 9.05 on the right and left, respectively. In one single-center observational study enrolling 238 patients who had undergone elective CABG, older age and higher rates of hypertension and DM were observed in patients with CAVI >9, which is similar to the results of our study [21].

One observational study demonstrated the positive correlation of arterial stiffness and the severity of coronary artery disease using arterial stiffness index, an index of vascular elasticity measured by oscillography [22]. Another single-center observational study, however, reported no correlation between CAVI and the number of diseased coronary arteries in patients who had undergone coronary artery bypass surgery [21]. Dyslipidemia is a known risk factor for arterial stiffness. In observational studies, CAVI values were significantly higher in dyslipidemic patients than in normolipidemic patients [23]. In our studies, more than 90 percent of the participants had dyslipidemia, and no difference was observed between the groups. This result means that our patients generally had multiple known cardiovascular risk factors, one of them being dyslipidemia, and the baseline prevalence of abnormal lipid profile among our patients was high. The effect of dyslipidemia on the arterial stiffness in both group was essentially equal. In addition, age is a known factor strongly correlated with the development of arterial stiffness. A 6-year difference was observed between groups with statistical significance in our study as well as in other prospective studies [21]. We performed multivariable logistic regression analysis and revealed that diabetes mellitus and MDA-oxLDL level were independent predictors of arterial stiffness.

The mechanism of arterial stiffness is composed mainly of endothelial dysfunction, change in extracellular matrix composition, calcification, impaired vascular smooth muscle cell relaxation, impaired nitric oxide bioactivity, inflammation, and multiple hormonals signaling pathways [1]. Ox-LDL is a marker of oxidative stress, and it is involved in atherosclerotic plaque formation, including endothelial dysfunction, foam cell formation, smooth muscle cell proliferation and migration, and extracellular matrix inflammation [8,9]. These processes may also play a role in arterial stiffness development. There is evidence demonstrating the correlation of oxidative stress and the mechanism of arterial stiffness. For instance, an in vitro study revealed that oxidized LDL stimulates collagen production in cultured arterial smooth muscle cells [24]. MDA-oxLDL has been used as an indicator of oxidative stress in previous research [25]. There is also evidence demonstrating correlation of oxidized LDL and aortic pulse wave velocity in older adults [17]. Previous evidence gave us a hint about the possible role of oxidative stress in the mechanism of arterial stiffness [17]. Serum MDA-oxLDL level had a positive correlation with the activity of rho-associated kinase pathways, which is involved in the mechanism of vasoconstriction and vascular stiffening [26,27]. Ox-LDL increases the expression of matrix metalloproteinases, which promotes the breakdown of extracellular matrix components and stimulates collagen synthesis in arterial smooth muscle cells, which may contribute to vascular remodeling and stiffness [1,28]. As a result, we believe that the oxidative stress is not only an important cause of the pathogenesis of atherosclerotic disease but also plays a role in the interaction of complex mechanisms of arterial stiffness. Further research is needed to determine exactly how it is involved in the disease process and whether therapy to reduce the oxidative stress would help to reduce the progress of arterial stiffness. Medication treatments—such as those for blood glucose, BP, and lipid control and lifestyle modification, including smoking cessations and exercise—have been demonstrated to lower CAVI [5,7]. A single-center observational study reported that pitavastatin may have an oxidative stress-reducing effect, resulting in the decrease in both CAVI and MDA-oxLDL level [29]. It has been reported in the results of a randomized control trial that rosuvastatin reduces serum MDA-LDL level after 12 weeks follow-up. The reduction of MDA-LDL level from baseline is greater in the high-dose group than in the control group [30].

The purpose of this study was to demonstrate the relationship between oxidative stress and arterial stiffness, and the result implies that there is a positive correlation. We believe that CAVI is a simple and effective tool to evaluate arterial stiffness, and the process of measurement would not be too troublesome for most patients. Thus, CAVI could be used to evaluate the treatment response or identify the patients at high risk and to adjust the treatment strategy accordingly. MDA-LDL is a helpful laboratory marker of oxidative stress; however, it might not be a convenient tool for use in a clinical scenario at present. Currently, we use MDA-LDL only for study purposes, and we do not suggest it should be measured in every post-CABG patient.

In this study, we used MDA-oxLDL as a marker of oxidative stress in our patients, and we found that MDA-oxLDL showed a significantly positive correlation with CAVI and was an independent predictor of arterial stiffness.

The relationship that we found between MDA-oxLDL and arterial stiffness might have some clinical significance. First, CAVI is a simple and effective tool to evaluate the extent of the arterial stiffness and to predict the risk of a cardiovascular event in patients with high CV risks. In addition, it could be used as a measurable goal for evaluating the effect of the treatment targeting the reduction of the arterial stiffness risk, including both pharmaceutical and lifestyle modification.

However, our study had several limitations. First, it was a cross-sectional observational study that analyzed a small sample size and was conducted in single center. Second, CAVI has been demonstrated to be positively correlated with the severity of coronary atherosclerosis [31,32]. In the present study, all patients had triple-vessel CAD. However, we did not implement a severity scoring system for the correlation of CAD severity with CAVI value, as it could not be objectively quantified and analyzed. This will be considered in our future research. Third, the study design did not enable us to derive any causative relationships from the statistical results. Therefore, further studies are needed to examine the role of the MDA-oxLDL in arterial stiffness development.

## 5. Conclusions

In a small cohort of patients with triple-vessel CAD who had undergone CABG, older age, DM, and MDA-oxLDL were found to be independent risk factors for arterial stiffness.

## Figures and Tables

**Table 1:** Baseline characteristics of the patients.

Characteristic	All Participants (*n* = 88)	Control Group (*n* = 41)	Arterial Stiffness Group (*n* = 47)	*p*-Value
Age (years)	64.49 ± 9.76	61.32 ± 10.62	67.26 ± 8.09	0.004 *
Height (cm)	163.95 ± 7.46	162.49 ± 8.00	165.22 ± 6.80	0.087
Body weight (kg)	72.63 ± 9.53	73.00 ± 9.09	72.31 ± 9.98	0.733
Body mass index (kg/m^2^)	27.02 ± 3.04	27.67 ± 2.92	26.45 ± 3.05	0.060
CABG operation duration (months)	27.62 (13.48–59.91)	35.23 (16.50–51.99)	26.17 (6.10–68.40)	0.707
Systolic blood pressure (mmHg)	135.86 ± 21.25	133.51 ± 20.62	137.91 ± 21.80	0.335
Diastolic blood pressure (mmHg)	74.30 ± 14.94	73.71 ± 15.74	74.81 ± 14.36	0.732
Right CAVI	8.79 ± 2.06	7.29 ± 1.53	10.11 ± 1.48	<0.001 *
Left CAVI	8.79 ± 2.03	7.26 ± 1.53	10.13 ± 1.36	<0.001 *
Right ABI	1.07 ± 0.10	1.07 ± 0.10	1.07 ± 0.09	0.864
Left ABI	1.05 ± 0.08	1.05 ± 0.08	1.04 ± 0.07	0.546
Total cholesterol (mg/dL)	152.79 ± 36.38	152.39 ± 29.98	160.68 ± 36.79	0.254
Triglyceride (mg/dL)	129.00 (100.00–164.00)	141.00 (98.50–190.00)	123.00 (100.00–158.00)	0.189
HDL-C (mg/dL)	43.10 ± 10.08	43.66 ± 8.40	43.04 ± 11.28	0.775
LDL-C (mg/dL)	95.47 ± 27.68	93.54 ± 27.50	99.28 ± 28.39	0.340
Fasting glucose (mg/dL)	105.50 (96.00–139.25)	105.00 (94.50–152.50)	106.00 (97.00–137.00)	0.735
Blood urea nitrogen (mg/dL)	15.00 (13.00–20.00)	16.00 (13.25–21.50)	15.00 (13.00–20.00)	0.654
Creatinine (mg/dL)	1.00 (0.80–1.20)	0.90 (0.80–1.20)	1.00 (0.80–1.20)	0.748
eGFR (mL/min)	73.04 ± 20.95	75.61 ± 21.82	73.76 ± 20.53	0.684
Cystatin C (mg/L)	1.02 (0.91–1.16)	0.98 (0.87–1.17)	1.03 (0.95–1.15)	0.143
Total calcium (mg/dL)	8.78 ± 0.44	8.76 ± 0.35	8.78 ± 0.46	0.862
Phosphorus (mg/dL)	3.53 ± 0.51	3.48 ± 0.47	3.57 ± 0.55	0.449
MDA-oxLDL (μg/mL)	1.63 (0.59–3.32)	1.10 (0.40–1.79)	2.60 (1.28–4.45)	<0.001 *
Male, *n* (%)	68 (73.3)	29 (70.7)	39 (83.0)	0.171
Diabetes mellitus, *n* (%)	41 (46.6)	14 (34.1)	27 (57.4)	0.029 *
Hypertension, *n* (%)	46 (52.3)	20 (48.8)	26 (55.3)	0.540
Dyslipidemia, n (%)	83 (94.3)	39 (95.1)	44 (93.6)	0.761
Statin used, *n* (%)	71 (80.7)	33 (80.5)	38 (80.9)	0.966
Fibrate used, *n* (%)	6 (6.8)	3 (7.3)	3 (6.4)	0.862

Normally distributed continuous variables are shown as mean ± standard deviation after analysis by Student’s *t*-test. Variables not normally distributed are shown as median and interquartile range after analysis by the Mann–Whitney U test. Categorical variables are presented as number (%) and analyzed by the chi-squared test. CABG, coronary artery bypass graft; CAVI, cardio-ankle vascular index; eGFR, estimated glomerular filtration rate; HDL-C, high-density lipoprotein cholesterol; LDL-C, low-density lipoprotein cholesterol; MDA-oxLDL, malondialdehyde-oxidized low-density lipoprotein. * *p* < 0.05 was considered statistically significant.

**Table 2:** Multivariate logistic regression analysis of the factors correlated with arterial stiffness among the 88 coronary artery bypass graft patients.

Variable	Odds Ratio	95% Confidence Interval	*p*-Value
MDA-oxLDL, 1 μg/mL	1.511	1.059–2.158	0.023 *
Diabetes mellitus, present	3.660	1.121–11.943	0.032 *
Age, 1 year	1.056	0.994–1.123	0.077
Female	0.935	0.137–6.366	0.945
Height, 1 cm	1.062	0.950–1.188	0.288
Body mass index, 1 kg/m^2^	0.879	0.727–1.062	0.182
Triglyceride, 1 mg/dL	0.993	0.984–1.002	0.141
Cystatin C, 1 mg/L	1.489	0.381–5.820	0.567

Malondialdehyde-oxidized low-density lipoprotein, MDA-oxLDL. * *p* < 0.05 was considered statistically significant in the forward multivariable logistic regression analysis (adopted factors: female, diabetes mellitus, age, triglyceride, height, body mass index, cystatin C, and MDA-oxLDL).

**Table 3:** Correlation between log-transformed MDA-oxLDL level and clinical variables.

Variable	Spearman’s Correlation Coefficient	*p*-Value
Left CAVI	0.338	0.003 *
Right CAVI	0.376	0.001 *
Log-CABG operation duration (months)	0.250	0.019 *
Left ABI	−0.142	0.186
Right ABI	0.021	0.848
Age (years)	0.135	0.211
BMI (kg/m^2^)	0.074	0.492
eGFR (mL/min)	−0.070	0.519
Log-Cystatin C (mg/L)	0.059	0.590
Log-Glucose (mg/dL)	0.066	0.542
TCH (mg/dL)	−0.005	0.964
Log-TG (mg/dL)	−0.051	0.639
LDL-C (mg/dL)	0.007	0.946
HDL-C (mg/dL)	0.027	0.806
SBP (mmHg)	−0.009	0.935
DBP (mmHg)	−0.020	0.850

Data of MDA-oxLDL, CABG operation duration, glucose, triglyceride, and cystatin C levels showed skewed distribution and, therefore, were log-transformed before analysis. Analysis of data was performed using the Spearman correlation analysis. MDA-oxLDL, malondialdehyde-oxidized low-density lipoprotein; CAVI, cardio-ankle vascular index; ABI, ankle–brachial index; BMI, body mass index; eGFR, estimated glomerular filtration rate; TCH, total cholesterol; TG, triglyceride; LDL-C, low-density lipoprotein cholesterol; DBP, diastolic blood pressure; SBP, systolic blood pressure. * *p* < 0.05 was considered statistically significant (2-tailed).

## Data Availability

Data are available from the corresponding author upon reasonable request.
